# Creating Interactions between Tissue-Engineered Skeletal Muscle and the Peripheral Nervous System

**DOI:** 10.1159/000443634

**Published:** 2016-11-09

**Authors:** Alec S.T. Smith, Samantha L. Passey, Neil R.W. Martin, Darren J. Player, Vivek Mudera, Linda Greensmith, Mark P. Lewis

**Affiliations:** ^a^Sobell Department of Motor Neuroscience and Movement Disorders, UCL Institute of Neurology, London, UK; ^b^MRC Centre for Neuromuscular Diseases, UCL Institute of Neurology, London, UK; ^c^Division of Surgery and Interventional Science, UCL Institute of Orthopaedics and Musculoskeletal Science, London, UK; ^d^Arthritis Research UK Centre for Sport, Exercise and Osteoarthritis, National Centre for Sport and Exercise Medicine (NCSEM) England, School of Sport, Exercise and Health Sciences, Loughborough University, Loughborough, UK; ^e^Department of Pharmacology and Therapeutics, University of Melbourne, Melbourne, Vic., Australia; ^f^Department of Bioengineering, University of Washington, Seattle, Wash., USA

**Keywords:** 3D tissue engineering, In vitro models, Motor neurons, Neuromuscular junctions, Skeletal muscle

## Abstract

Effective models of mammalian tissues must allow and encourage physiologically (mimetic) correct interactions between co-cultured cell types in order to produce culture microenvironments as similar as possible to those that would normally occur in vivo. In the case of skeletal muscle, the development of such a culture model, integrating multiple relevant cell types within a biomimetic scaffold, would be of significant benefit for investigations into the development, functional performance, and pathophysiology of skeletal muscle tissue. Although some work has been published regarding the behaviour of in vitro muscle models co-cultured with organotypic slices of CNS tissue or with stem cell-derived neurospheres, little investigation has so far been made regarding the potential to maintain isolated motor neurons within a 3D biomimetic skeletal muscle culture platform. Here, we review the current state of the art for engineering neuromuscular contacts in vitro and provide original data detailing the development of a 3D collagen-based model for the co-culture of primary muscle cells and motor neurons. The devised culture system promotes increased myoblast differentiation, forming arrays of parallel, aligned myotubes on which areas of nerve-muscle contact can be detected by immunostaining for pre- and post-synaptic proteins. Quantitative RT-PCR results indicate that motor neuron presence has a positive effect on myotube maturation, suggesting neural incorporation influences muscle development and maturation in vitro. The importance of this work is discussed in relation to other published neuromuscular co-culture platforms along with possible future directions for the field.

**Table:** Abbreviations used in this paper

AChR	acetylcholine receptor
AChRε	acetylcholine receptor ε-subunit
ALS	amyotrophic lateral sclerosis
BSA	bovine serum albumin
DIV	days in vitro
DMEM	Dulbecco's modified Eagle's medium
ESC	embryonic stem cell
HBSS	Hanks' balanced saline solution
iPSC	induced pluripotent stem cell
MAP-2	microtubule-associated protein-2
MDC	muscle-derived cell
MYH1	myosin heavy chain 1 (adult fast isoform)
MYH3	myosin heavy chain 3 (embryonic isoform)
MYH8	myosin heavy chain 8 (neonatal isoform)
NGS	normal goat serum
NMJ	neuromuscular junction
P1	postnatal day 1
P/S	penicillin/streptomycin
PBS	phosphate-buffered saline
RPIIB	RNA polymerase II β-subunit
RT-PCR	reverse transcription polymerase chain reaction
SV-2	synaptic vesicle protein-2
TBS	Tris-buffered saline
TGF-β	transforming growth factor-β

## Introduction

### Engineering in vitro Neuromuscular Junctions

In seeking to engineer in vitro culture models that accurately recapitulate the physiology and function of in vivo tissues, there is a critical need to develop platforms in which the principal cell types can interact correctly. Therefore, in order to model skeletal muscle effectively, the engineered tissue should be able to generate biologically accurate representations of the vascular interface as well as the myotendinous and neuromuscular junctions (NMJs). The successful development of 3D skeletal muscle models which enable interaction with these supporting tissues and structures would lead to the development of mature and biologically accurate model systems for musculoskeletal disease modelling, drug screening, and developmental or mechanistic studies [Larkin et al., 2006b]. Previous publications have focused on the development of both vascular [Levenberg et al., 2005; Borschel et al., 2006; Hadjipanayi et al., 2011; Juhas et al., 2014] and myotendinous interfaces [Larkin et al., 2006a; Kostrominova et al., 2009] within existing in vitro skeletal muscle models. However, the development of robust, fully functional NMJs in vitro has yet to be demonstrated.

### Motor Neuron-Myotube Co-Cultures

Although co-culture of nerve and muscle tissue was first attempted over a century ago [Harrison et al., 1907], it is only relatively recently that significant methodological advances have been made. Conventional cell culture techniques have shown that motor neurons are capable of eliciting end-plate potentials in co-cultured myotubes. Dual-patch analysis of mouse embryonic stem cell (ESC)-derived motor neurons and C2C12 myotubes co-cultured at low density has shown that electrical activation of neurons will trigger post-synaptic potentials in associated muscle fibres in vitro [Umbach et al., 2012]. Similarly, functional analysis of primary rat skeletal muscle myotubes on microscale cantilevers has demonstrated that activation of co-cultured primary rat embryonic ventral horn motor neurons through application of glutamate will induce contractile activity in underlying myotubes. Such activation has also been shown to be subsequently blocked through addition of the acetylcholine receptor (AChR) inhibitor D-tubocurarine [Smith et al., 2013]. Co-cultures of human cells have even provided evidence for functional contact between these cell types, again though blockage of contractile activity through treatment with D-tubocurarine [Guo et al., 2011]. In addition, modified Campenot chambers have been developed to separate neuron and muscle cell bodies in order to better model the compartmentalised nature of the in vivo peripheral nervous system [Southam et al., 2013]. Microfluidic chambers have also been developed, in which fluids are manipulated at the submillimetre scale [Taylor et al., 2006]. Using this approach, multicompartment culture chambers can be produced which not only enable neuronal processes and muscle cells to be fluidically isolated from cell bodies, but also allows the cells to be monitored by live cell imaging, as well as phase, differential interference contrast, and fluorescence microscopy [Taylor et al., 2006].

These systems provide valuable proof of concept for the generation of functional synapses between motor neurons and muscle cell types in vitro, and similar systems have been used to investigate acetylcholinesterase production and cycling at the NMJ [Jevsek et al., 2004; Mis et al., 2005], highlighting how such models can be used for mechanistic evaluation of neuromuscular synaptic function. However, immunocytochemical data from these studies indicate a relatively immature state of development in these in vitro NMJs. Cultured synapses typically lack an invaginated, ‘pretzel-like’ structure in the post-synaptic membrane, have low AChR cluster density, display a paucity of specialised subsynaptic nuclei, and rarely promote pre-synaptic terminal development and specialisation. These structural and molecular motifs are well-established hallmarks of mature NMJs [Sanes and Lichtman, 1999, 2001] and are necessary for accurate modelling of these synapses for drug screening, disease modelling, and fundamental mechanistic studies. Recently, the development of more structurally mature NMJs in conventional co-culture has been reported [Chipman et al., 2014]. Mouse ESC-derived motor neurons encased in embryoid bodies and co-cultured with embryonic chick myotubes were shown to develop advanced end-plate morphologies reminiscent of NMJs in vivo, demonstrating that the achievement of such structures is possible in vitro. Furthermore, this model was used to characterise synaptic development in neural cell adhesion molecule (NCAM)-/- mutant cells, providing valuable data regarding the role of NCAM in modulating synaptic vesicle endocytosis to influence neuromuscular synaptogenesis. Results such as these highlight the potential utility of advanced NMJ models for conducting mechanistic evaluation of synaptic development, and even for drug screening or disease modelling applications.

### 3D Muscle Constructs for Motor Neuron-Myotube Co-Culture Studies

Culturing of skeletal muscle cells in a 3D environment through integration with an exogenous matrix material has been shown to significantly improve the maturation state of cultured myotubes, with respect to levels of muscle cell fusion, contractile force output, sarcomeric development, cellular organisation, and up-regulation of muscle-specific myogenic regulatory factors [Vandenburgh et al., 1999; Dennis et al., 2001; Powell et al., 2002; Mudera et al., 2010; Hinds et al., 2011; Smith et al., 2012; Juhas et al., 2014; Madden et al., 2015]. Given the immature nature of many in vitro NMJ culture systems, it seems plausible that improvements in cellular maturation may promote the development of more robust and developmentally advanced synapses in such culture models.

Co-culture of 3D, fibrin-based, skeletal muscle constructs with organotypic neuronal slices has previously been shown to promote the up-regulation of important NMJ markers such as the AChR ε-subunit (AChRε) and the dihydropyridine receptor Trisk 51 [Bach et al., 2003]. Likewise, myosin heavy chain expression patterns appear to progress from neonatal isoforms towards a more developmentally mature phenotype in such constructs [Larkin et al., 2006b]. Myotubes co-cultured with neuronal tissue in 3D exhibit spontaneous contractions that can be blocked by treatment with depolarising and non-depolarising muscle relaxants, as well as significant increases in peak twitch and tetanic contractile responses to timed electrical stimuli. Similarly, fibrin-based constructs seeded with muscle-derived cells (MDCs) and implanted in close proximity to the transected femoral nerve of adult rats have demonstrated far greater contractile function when compared with control cultures following 4 weeks in situ [Dhawan et al., 2007]. Recently, work has been published demonstrating the production of a co-culture system supporting synaptic contact between aligned myotubes and motor neuron neurospheres derived from mouse neural stem cells [Morimoto et al., 2013]. These cultures were also able to promote improved contractile properties compared with muscle-only controls and spontaneous twitch activity that was interrupted by treatment with the AChR blocker curare.

### Limitations of Current Models

Such studies demonstrate the improvement in MDC maturation that can be brought about by the inclusion of neuronal tissue within a 3D skeletal muscle model and highlight the corresponding improvement in contractile function that this generates. However, the work to date has still failed to promote the development of structurally and functionally mature NMJs as would be seen in vivo. Moreover, the majority of work to date focuses on the use of either organotypic slices of central nervous system tissue [Bach et al., 2003; Larkin et al., 2006b], transected nerve tissue [Dhawan et al., 2007], or aggregated and ill-defined neurospheres [Morimoto et al., 2013] to provide the neural input for the seeded muscle cells. Consequently, there remains some ambiguity as to whether or not isolated motor neurons are capable of surviving and interacting correctly with MDCs within a 3D culture environment, and whether the other cell types found in neurosphere models can indeed enhance/interact with neurons to support maturation.

The development of induced pluripotent stem cell (iPSC)-derived motor neurons capable of accurately modelling peripheral neuropathies [Dimos et al., 2008; Bilican et al., 2012; Chestkov et al., 2014; Demestre et al., 2015] suggests that the development of 3D biomimetic culture platforms for the study of diseases such as amyotrophic lateral sclerosis (ALS) may be possible in the near future [Richard and Maragakis, 2014]. However, the establishment of such model systems is predicated on the development of culture protocols for maintaining dissociated single neurons with engineered skeletal muscle tissue in the medium to long term. Furthermore, the published work on this subject has so far been limited to investigation of fibrin- and Matrigel-based constructs [Bach et al., 2003; Larkin et al., 2006b; Dhawan et al., 2007; Morimoto et al., 2013]. At present, there are no published data regarding the ability to promote neuromuscular interaction within alternative in vitro matrices. Critical appraisal of the suitability of a given 3D system for studying neuromuscular interaction in vitro necessitates the dissemination of data obtained from a variety of suitable matrices in order to discern which scaffold possesses the most appropriate characteristics for continued investigation. Given the high concentration of collagen in the extracellular matrix of native skeletal muscle [Gillies et al., 2014], it is sensible to assume that collagen matrices would represent a suitable in vitro scaffold for use in engineering more biomimetic skeletal muscle tissues. Evaluation of the capacity of collagen matrices to promote synaptic development in motor neuron-myotube co-cultures is therefore worthy of investigation and subsequent comparison to similar data obtained in published fibrin and Matrigel systems.

### Analysis of Isolated Motor Neuron Interaction with a Tissue-Engineered Biomimetic 3D Skeletal Muscle Model

As part of this study, we present the development of a 3D, collagen-based, co-culture system for the maintenance of aligned, primary MDCs and ventral horn motor neurons. This work is based on the development of a well-established long-term culture model for maintaining primary skeletal muscle myotubes in 3D constructs [Smith et al., 2012]. The stability of this culture system in turn makes it a strong candidate for enabling the generation of long-term 3D nerve-muscle co-cultures in order to analyse synaptic development in vitro. Additionally, the developed model is amenable to mechanical loading techniques [Player et al., 2014], which will enable subsequent studies into the role of mechanical cues in driving neuromuscular development.

The ability for synaptic contacts to form within this model and the positive effect of the neuronal presence on the MDC phenotype were confirmed and quantified. The presented data validate the potential for isolated single neurons to survive and influence myotube development in 3D co-culture, paving the way for the integration of isolated stem cell-derived motor neurons with biomimetic skeletal muscle cultures. Such culture systems will be invaluable for furthering our understanding of disease pathogenesis as well as for providing a reliable pre-clinical test bed for the future development of novel therapeutics.

## Materials and Methods

### Cell Culture

#### Establishment of Primary MDC Cultures

On postnatal day 1 (P1) Sprague-Dawley rat pups were euthanised by cervical dislocation, in accordance with the code of practice for the humane killing of animals under Schedule 1 of the Animals (Scientific Procedures) Act 1986. The protocol for the isolation of MDCs, as well as the verification of a stable myogenic population present among the isolated cells, has been previously described in detail [Smith et al., 2012]. Briefly, following removal of the skin, the hindlimbs were transferred to a Petri dish containing phosphate-buffered saline (PBS) solution (Sigma-Aldrich, Poole, UK) supplemented with 2% penicillin (100 units/ml)/streptomycin (100 μg/ml) (P/S) (Gibco/Invitrogen, Paisley, UK). The muscle tissue was separated from the bone before both bone and cartilage were removed from the Petri dish and discarded. The muscle tissue fragments were then suspended in 0.1% collagenase (Gibco/Invitrogen) in PBS and incubated in a 37°C shaking incubator set to 300 rpm for 50 min, or until the tissue was fully digested.

The resulting cell solution was passed through a 100- and then a 40-μm mesh filter (BD Biosciences) in order to remove any debris or undigested tissue fragments, before being spun at 450 *g* for 10 min. The cells were then re-suspended in 500 μl standard growth medium (medium 1; see online suppl. table 1; for all online suppl. material, see www.karger.com/doi/10.1159/000443634) consisting of high-glucose Dulbecco's modified Eagle's medium (DMEM) (Gibco/Invitrogen) supplemented with 20% fetal calf serum (PAA, Yeovil, UK) and 1% P/S.

Isolated MDCs for 2D experiments were seeded onto gelatin-coated, 13-mm, sterile, glass coverslips at a density of 50,000 cells/cm^2^. Cultures were maintained in medium 1 until they reached confluency. At this point, the medium was replaced with a differentiation medium (medium 2; see online suppl. table 1) in order to promote fusion of the myogenic cells. Agrin and Wnt3 were added to the medium since they have been found to significantly improve levels of post-synaptic differentiation in cultured myotubes [Henriquez and Salinas, 2012]. The ability for these proteins to improve the number of AChR clusters formed on cultured myotubes when included in the feeding medium, as reported previously, was verified during initial optimisation of culture parameters (online suppl. fig. 1). Cultures were maintained in medium 2 for 3 days (for myoblast purity assessment) or 10 days (for myotube fusion assessment) before being fixed and prepared for post-hoc analysis.

#### Establishment of Primary Mixed Ventral Horn Cultures

Primary motor neurons were isolated from rat embryos at gestational age E14 using a method modified from that described by Henderson et al. [1995] and Kalmar and Greensmith [2009]. Pregnant Sprague-Dawley females were euthanised by exposure to a rising concentration of carbon dioxide gas, in accordance with the code of practice for the humane killing of animals under Schedule 1 of the Animals (Scientific Procedures) Act 1986.

Embryos were removed following hysterectomy and transferred to a Petri dish containing Hanks’ balanced saline solution (HBSS) (Sigma-Aldrich) supplemented with 2% P/S. Spinal cords were separated from the surrounding tissue and the meninges carefully removed. The dorsal horn was then cut away from the ventral portion of the spinal cord and discarded. The ventral horns from individual embryos were pooled and incubated in a 0.025% trypsin solution (type XII-S) (Sigma-Aldrich) in HBSS for 10 min. They were then transferred to a fresh solution containing 800 μl L-15 medium (Gibco/Invitrogen), 100 μl 4% bovine serum albumin (BSA) (Sigma-Aldrich) and 100 μl DNase (1 mg/ml stock) (Sigma-Aldrich). The spinal cords were agitated vigorously until they had disaggregated and were then triturated using a P1000 tip and left to settle. After 2 min, the solution was transferred to a 15-ml centrifuge tube (care was taken to avoid transferring any undissociated fragments). This process was repeated twice and the three supernatants were pooled before being spun through a 1-ml 4% BSA cushion for 5 min at 370 *g*.

Once the supernatant had been removed, the pellet was re-suspended in medium 2 and plated onto polyornithine- and laminin-treated, 13-mm, sterile glass coverslips at a density of 25,000 cells/cm^2^. Cultures were maintained in vitro for 7 days before being prepared for analysis. During this culture period, the feeding medium was changed every 2–3 days.

#### Establishment and Maintenance of 3D Primary MDC Cultures

**Fig. 1:**
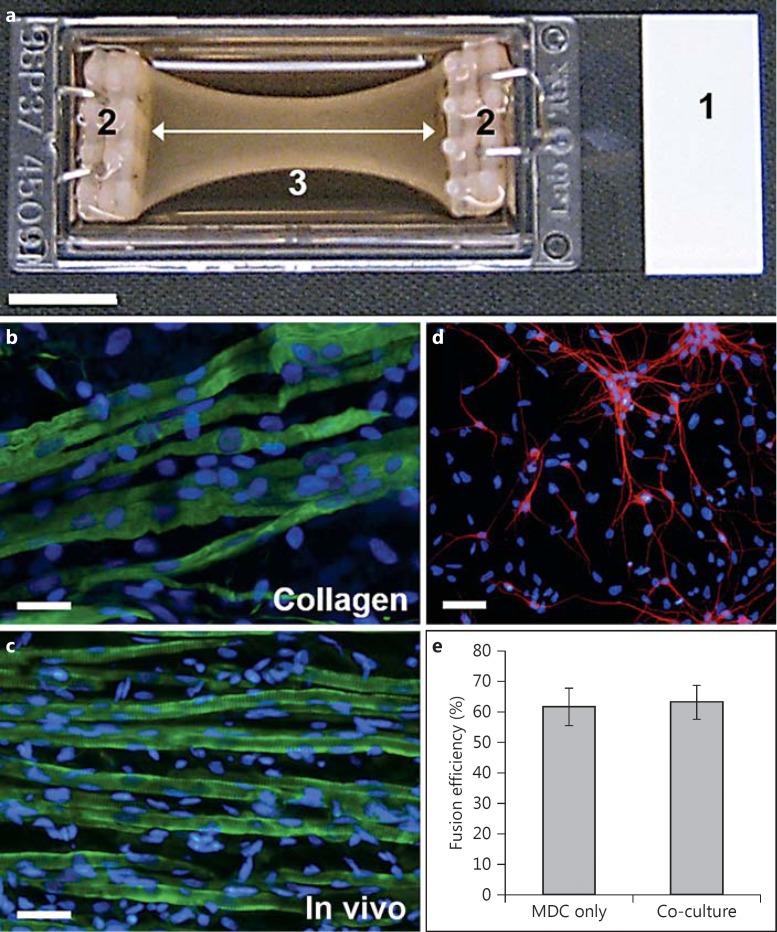
3D co-culture platform and cell population characterization. **a** A chamber slide (1) fitted with custom-built flotation bars at either end (2). A cell-laden collagen solution is allowed to gel between the flotation bars. The collagen gel is then detached from the edges of the chamber slide so that it floats in the culture medium. Cell-mediated matrix compaction leads to the generation of isometric strain between the flotation bars (white arrow). Compaction of the collagen gel over time produces the characteristic bowing illustrated (3). Scale bar = 10 mm. **b** Image of MDCs maintained in this culture platform for 2 weeks and stained for desmin (green) and nuclei (blue). Note the parallel alignment of the developing myotubes. Scale bar = 20 μm. **c** Image of a muscle tissue section taken from a P1 rat pup, cryosectioned, and stained for desmin (green) and nuclei (blue). Scale bar = 20 μm. **d** Image of ventral horn motor neurons maintained on glass coverslips for 7 days before being stained for MAP-2 (red) and nuclei (blue). Scale bar = 40 μm. **e** Comparison of fusion efficiency in MDC-only 3D constructs versus motor neuron-muscle co-cultures. p = 0.86.

The protocol for establishing 3D cultures of aligned myotubes has been previously described in detail [Smith et al., 2012] and was adapted from methods developed for use with the culture force monitor system [Brady et al., 2008; Mudera et al., 2010]. Type 1 rat tail collagen (2.6 ml at 2.05 mg/ml in 0.1 M acetic acid; First Link, Wolverhampton, UK) was mixed with 300 µl 10× minimal essential medium (Gibco/Invitrogen). 5 M NaOH (VWR) was then added dropwise until a colour change from yellow to pink was observed. This solution was then supplemented with 300 µl of medium 1 containing 15 × 10^6^ primary rat MDCs. The cell-collagen solution was pipetted into a prepared single-well chamber slide (Nunc, New York, N.Y., USA) containing a collagen-coated flotation bar fixed at either end (fig. 1a). The construct was incubated for 30 min or until the collagen gel had set. After incubation, the construct was cut away from the sides of the chamber slide using a sterile needle and floated in medium 1. Care was taken to ensure the construct was fully detached from all surfaces except the flotation bars. After 4 days in vitro (DIV), medium 1 was replaced with medium 2 for the remainder of the culture period. All media were replaced daily.

#### Establishment and Maintenance of 3D Primary MDC-Motor Neuron Co-Cultures

3D muscle constructs were established as described aboveand maintained in medium 1 for 4 days. At this point, medium 1 was replaced with medium 2 and 1 × 10^6^ mixed ventral horn cells, suspended in 100 µl medium 2, were pipetted directly onto the upper surface of the collagen gel. Constructs were returned to the incubator and maintained for a further 14 days before being prepared for analysis. Media were changed daily throughout the culture period.

#### Immunocytochemistry

Cultures were fixed with an ice-cold methanol/acetone solution. 3D constructs (and in vivo tissue sample controls from P1 rat hindlimbs) were then dehydrated in a sucrose solution, frozen in OCT medium (Tissue-Tek/Sakura Finetek Europe, Alphen aan den Rijn, The Netherlands) and sectioned using a Bright™ cryostat; 30-µm sections were collected on poly-L-lysine-coated slides (VWR) and air-dried for 1 h before staining. 3D and 2D samples were permeabilised using 1× Tris (0.5 M) buffered saline solution (TBS) [+ 5% normal goat serum (NGS) and 0.2% Triton X-100] for 1 h. They were then incubated overnight at 4°C with the chosen primary antibody diluted in TBS (+ 2% NGS and 0.2% Triton X-100). After overnight incubation, the samples were washed thoroughly in TBS before being treated with a secondary antibody, again diluted in TBS (+ 2% NGS and 0.2% Triton X-100). Nuclei were identified using the fluorescent minor-groove DNA-binding probe DAPI (4,6-diamidino-2-phenylindole; 1.0 ng/ml; Sigma-Aldrich), which was incorporated into the secondary antibody incubation stage. The samples were incubated for 3 h at room temperature in a darkened chamber before being dried and mounted with glass coverslips (VWR) using a drop of Mowiol mounting medium. 2D controls were dried and affixed to glass microscope slides, again using a drop of Mowiol mounting medium. Cells were visualised using a Zeiss LSM510 Meta confocal microscope.

Immunocytochemical analysis of 2D and 3D cultures was carried out using antibodies to a number of markers of muscle and motor axons, including antibodies to desmin (Dako, Ely, UK; 1:200), microtubule-associated protein-2 (MAP-2; Millipore, Watford, UK; 1:1,000), neurofilament (DSHB, Iowa City, Iowa, USA; 1:10), and synaptic vesicle protein-2 (SV-2; DSHB; 1:10), and markers of myotubes, neurons, axons, and pre-synaptic terminals, respectively. Texas-red-conjugated α-bungarotoxin (Sigma-Aldrich; 1:1,000) was also used to label AChR clusters on the post-synaptic myotube membrane. The secondary antibodies used were Alexa Fluor 488 donkey anti-mouse IgG (Gibco/Invitrogen) and Alexa Fuor 594 donkey anti-rabbit IgG (Gibco/Invitrogen), both diluted 1:200.

#### Image Analysis

MDCs and mixed ventral horn cells were stained for desmin and MAP-2, respectively. Nuclei were visualised by staining with the DNA-binding probe DAPI, and the number of nuclei present was used to calculate the percentage positivity of myoblasts and motor neurons in their respective cultures.

A myoblast was identified as any mononuclear desmin-positive cell, while a myotube was defined as a desmin-positive cell with at least 3 nuclei. Motor neurons were defined as MAP-2-positive cells with a cell body greater than 15 μm in diameter with at least 3 neuritic processes.

Cultures stained using Texas-red-conjugated α-bungarotoxin were analysed for clusters of AChR. An AChR cluster was classified as foci of red staining separate and distinct from others nearby and clearly distinguishable above background staining levels. Macroscopic images of 3D constructs were taken using a Canon PowerShot A460 5.0 MP digital camera and analysed using ImageJ software (National Institutes of Health, USA).

#### Quantitative One Step Reverse-Transcription Polymerase Chain Reaction

3D constructs were homogenised using an IKA Ultra-Turrax T10 homogeniser (Fisher Scientific, UK) in TRIzol reagent (Sigma-Aldrich). RNA was extracted according to the manufacturer's instructions and re-suspended in RNA storage solution (Ambion, Life Technologies, Paisley, UK). RNA quantity was determined spectrophotometrically using a Nanodrop 2000c.

Quantitative one step reverse-transcription polymerase chain reaction (RT-PCR) was performed using a Qiagen Rotor-Gene Q and SYBR green RT-PCR (Qiagen, Crawley, UK) one-step chemistry kit; 70 ng of total RNA were added per PCR, and reactions were performed in triplicate. The thermal cycling protocol consisted of a 10-min reverse transcription step at 50°C, followed by 40 cycles of 10 s at 95°C (denaturation) and 30 s at 60°C (annealing and extension). RT-PCR data were analysed by relative quantification, using the 2^-ΔΔCT^ method [Livak and Schmittgen, 2001]. Gene expression in both MDC-only and co-culture samples were normalised to RNA polymerase II β-subunit (RPIIB) and expressed relative to MDC-only constructs. Primers sequences used are provided in online supplementary table 2.

Following RT-PCR, the products of these reactions were separated using horizontal agarose gel electrophoresis. PCR products were excised and purified using the QIAquick gel extraction kit according to the manufacturer's protocol (Qiagen). They were then sent for sequencing at Geneservice (London, UK). Sequences were analysed using FinchTV software and compared to the known DNA sequence from an online database of the respective gene (www.ncbi.nlm.nih.gov) in order to confirm the validity of the primers used for amplifying sequences from the target gene.

#### Statistical Analysis

All experiments were repeated at least 3 times using cultures prepared on different days from pups of different litters. For all relevant experiments, statistical significance between 2 groups (e.g. 3D cultures ± motor neurons) was assessed using t tests in SigmaStat (version 2.03; Systat Software, Erkrath, Germany). For quantitative RT-PCR analysis, mRNA levels in co-culture were expressed relative to MDC-only control values. Since all MDC-only values were therefore equal to 1, a one-sample t test was used to evaluate whether relative gene expression levels in co-culture were significantly different from 1. All values detailed in this paper are expressed as means ± SEM. Significance was set at p < 0.05.

## Results

### MDC Differentiation Using Defined Media

Since MDCs and motor neurons exhibit distinct nutrient requirements in order to sustain growth and promote differentiation in vitro, medium 2 was developed specifically for the maintenance of these cell types in a 3D co-culture system. It was adapted from a medium used to maintain motor neurons in vitro and contains all the necessary factors to facilitate MDC differentiation. To verify the ability of this medium to promote myoblast differentiation, confluent MDCs were maintained in medium 2 for 3 days. The cultured cells were then fixed, stained for desmin, and assessed for fusion efficiency [the number of myogenic nuclei incorporated into myotubes (calculated as a percentage of the total number of myogenic nuclei present in culture)].

MDCs cultured in medium 2 on glass coverslips exhibited a fusion efficiency of 71.22% (±2.76), indicating that almost 3 quarters of all myogenic cells in culture were able to form myotubes when maintained in this medium. Our previous work with MDC-only cultures found that conventional differentiation medium, consisting of low-percentage horse serum in high-glucose DMEM, promoted fusion efficiencies of 61.46% (±1.16) [Smith et al., 2012]. The results obtained for cultures maintained in medium 2 were significantly greater than those obtained when the cells were exposed to a more conventional muscle differentiation medium, as detailed in our previous publication (p < 0.05). This observation indicates that the presence of the neuronal growth factors used in medium 2 may have had a beneficial effect on myoblast differentiation. The high level of myoblast fusion, coupled with the substantial motor neuron presence in mixed ventral horn cultures, demonstrates the suitability of medium 2 for the maintenance of MDC-motor neuron co-cultures. It should be stated that other factors relating to myoblast metabolism, growth, and/or survival may have been negatively affected by addition of neuronal factors to the medium, but were not measured in this study.

### MDC Differentiation in 3D Constructs Cultured with and without Motor Neurons

MDCs, maintained in medium 2, were found to differentiate into uniaxially arranged myotubes (fig. 1b) that mimicked the physiological architecture of native skeletal muscle when cultured within the described 3D platform (fig. 1c). Fusion efficiencies of MDCs in 3D co-culture and MDC-only controls were examined after 18 DIV (14 DIV in co-culture), in order to determine whether or not co-culture with primary motor neurons had any effect on the ability of primary MDCs to differentiate into multinuclear myotubes. Analysis showed that the fusion efficiencies of MDCs in mono- and co-culture were 61.64% (±6.04) and 63.17% (±5.52), respectively (fig. 1e). These values were not significantly different from each other (p = 0.86), indicating that motor neuron presence had no negative effect on MDC ability to fuse in 3D co-culture.

### Characterisation of Mixed Ventral Horn Cultures

Motor neurons from mixed ventral horn populations (fig. 1d), maintained in medium 2, were found to constitute 34.85% (±2.15) of all cells in culture following 7 DIV. This result is in line with data published previously regarding the size of the motor neuron fraction from unsorted ventral horn cells from our group [Kalmar and Greensmith, 2009] as well others [Bloch-Gallego et al., 1991], indicating the suitability of the described protocol and medium composition for obtaining and culturing primary motor neurons in vitro.

### Motor Neuron Survival, Neurite Outgrowth, and NMJ Formation in 3D Co-Culture

Motor neuron survival following 14 days in 3D co-culture with primary MDCs was examined by immunostaining for the neuronal marker MAP-2. The inherent heterogeneity of the primary cells employed in this co-culture system made distinguishing ventral horn-derived non-neuronal nuclei from non-myogenic nuclei present in MDC cultures problematic. However, MAP-2 immunostaining indicated that a substantial number of neurons survived on the construct surfaces to the end of the designated culture period. No MAP-2-positive staining was observed on MDC-only control cultures, indicating that the MAP-2 staining observed in the co-cultures was not due to non-specific binding or background fluorescence.

**Fig. 2:**
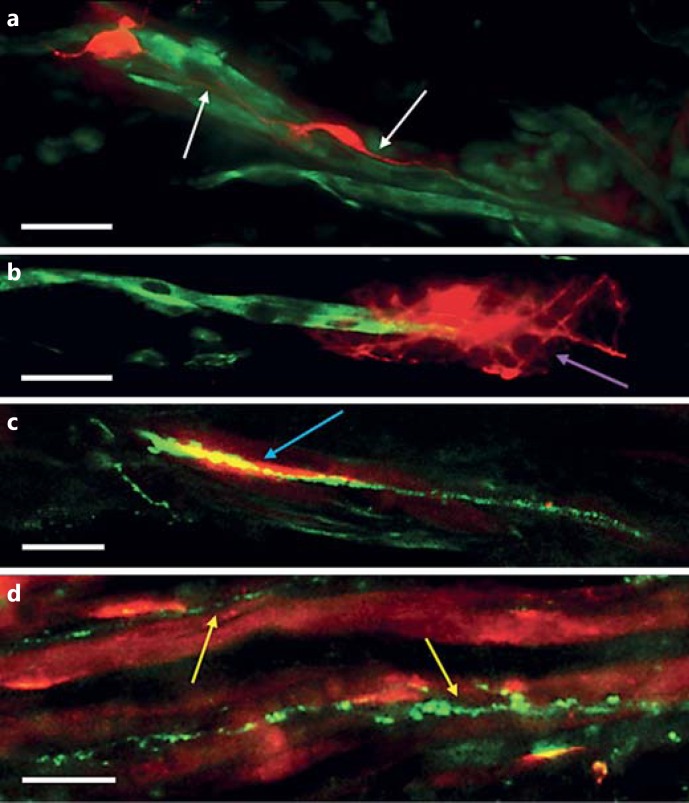
Neurite development and synaptic contact within 3D collagen-based co-culture constructs. Longitudinal slices (30 µm) were taken from 3D constructs for immunostaining and imaging. **a**, **b** Sections were stained for desmin (green) and MAP-2 (red). Scale bars = 20 μm. **c**, **d** Sections were stained for SV-2 (green) and AChRs (red). Scale bars = 10 μm. **a** Neurites were typically seen tracking along parallel, and in close proximity to, underlying myotubes (white arrows). **b** Occasionally, neurites were also found wrapped around cultured myotubes (purple arrow). **c** Pre- and post-synaptic co-localisation (blue arrow) was observed at a frequency of 4.24/mm^2^. **d** SV-2 tracks followed underlying myotube orientations (yellow arrows) indicating the path of neurite development and axonal transport of synaptic proteins from the cell bodies toward the developing growth cone.

Confocal analysis of 3D tissue sections revealed that MAP-2-positive neurites had infiltrated into the body of the muscle constructs (fig. 2a, b), highlighting the ability of cultured motor neurons to promote growth cone extension through the collagen matrix. All neuritic extensions were detected within the first 100 μm of the 3D tissue across all cultures examined (n = 3), suggesting either that the axons were unable to penetrate deeper into the construct or that cues from the myotubes at the surface promoted neurite localisation in the periphery of the scaffold matrix. Neurites were typically seen running parallel, and in close proximity to, the underlying myotubes (fig. 2a; arrows) but were also observed wrapping themselves around these multinuclear desmin-positive cells (fig. 2b; arrow).

Co-localisation of AChRs stained with α-bungarotoxin and the pre-synaptic marker synaptic vesicle protein 2 (SV-2) suggests the close association of pre- and post-synaptic terminals in this model (fig. 2c, d). These structures mirrored those observed at functional NMJs in vivo and provide evidence for the formation of putative NMJ-like structures in these 3D collagen-based co-culture constructs. Strands of SV-2-positive staining were also consistently observed running in close proximity to underlying myotubes, indicating the path of developing neurites through the collagen construct. Incidences of pre- and post-synaptic marker co-localisation occurred at a frequency of 4.24/mm^2^ (±2.33) in the examined co-cultures (n = 4). In MDC-only control cultures, no positive SV-2 staining was present, and so no co-localisation with AChR clusters was observed.

### Gene Expression Analysis in 3D Constructs Cultured with and without Motor Neurons

Quantitative RT-PCR results indicated the expression of multiple markers of skeletal muscle maturation present in both co-cultures and MDC-only constructs. Messenger RNA transcripts for both developmental myosin heavy chain (MYH; MYH3 and MYH8) and adult (MYH1) isoforms were detected in all cultures examined (n = 3), along with transcripts for troponin T1, indicating a transcriptional drive towards the generation of the functional contractile machinery within the cultured myotubes. In these experiments, the addition of motor neurons was associated with a significant up-regulation of MYH3 (p < 0.01) and MYH8 (p < 0.03) mRNA expression compared with MDC-only controls. An increase of similar magnitude in MYH1 expression was also observed, although this did not reach significance (p = 0.09). A significant 3-fold up-regulation (p < 0.05) in troponin T1 expression was also observed in co-culture constructs compared with MDC-only controls. The up-regulated MYH and troponin data indicate that the neuronal presence had a positive effect on the maturation of contractile machinery expression in the underlying myotubes in these cultures.

**Fig. 3:**
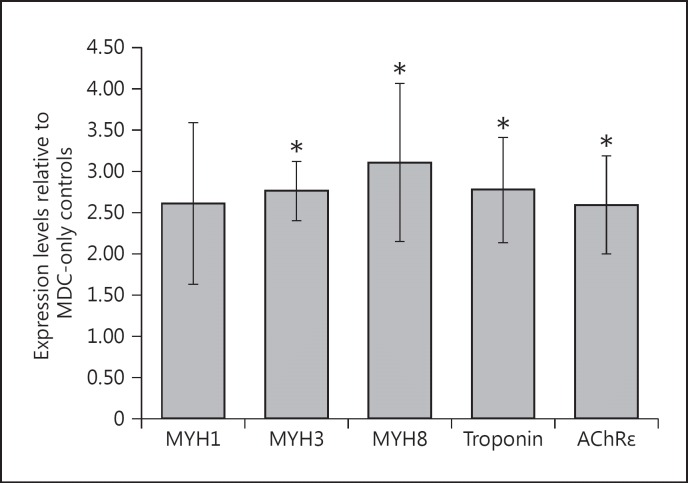
Gene expression changes in motor neuron-muscle co-cultures. Fold change in mRNA expression levels for genes encoding markers of myotube maturation and post-synaptic membrane development, measured using quantitative RT-PCR. Expression levels of MYH1 (adult fast isoform), MYH3 (embryonic isoform), and MYH8 (neonatal isoform), as well as troponin T1 and AChRε were quantified and expressed relative to levels recorded for 3D constructs without motor neurons at equivalent time points. In all cases, C_T_ values were normalised to an internal housekeeping gene (RPIIB) before subsequent analysis. For all genes examined, with the exception of MYH1, values were found to be significantly greater than those recorded for MDC-only controls (* p < 0.05). n = 3. Error bars = SEM.

AChRε subunit expression was significantly up-regulated in co-culture constructs compared with MDC-only controls (p < 0.05), indicating a maturation of the AChR assemblies present in the membranes of myotubes co-cultured with motor neurons. These data support previously discussed immunochemical analysis and provide further evidence for the formation of putative synaptic contact within these co-culture constructs (fig. 3).

### Macroscopic Gel Contraction in 3D Constructs Cultured with and without Motor Neurons

**Fig. 4:**
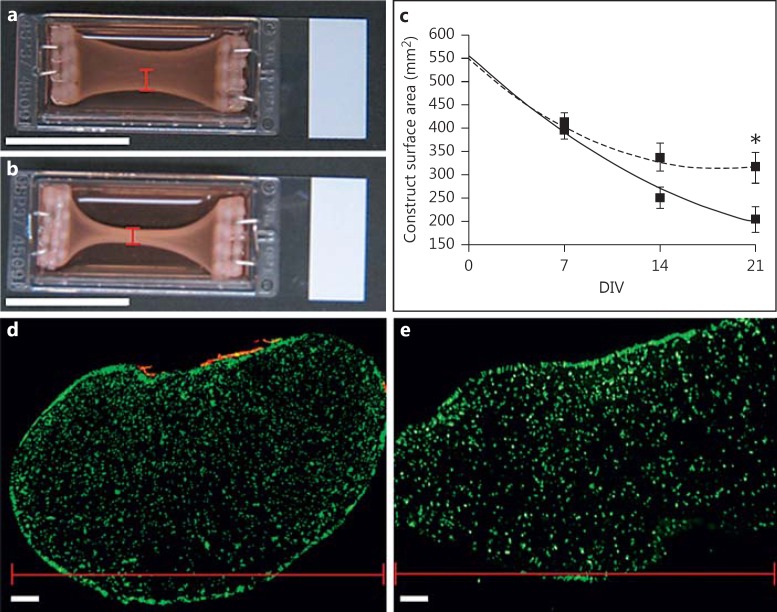
The effect of motor neuron presence on matrix compaction. **a**, **b** Macroscopic images of collagen constructs seeded with 5 × 10^6^ MDCs/ml following 21 DIV. Scale bars = 25 mm. **a** MDC-only control. **b** Motor neuron-myotube co-culture. **c** Graph detailing the measured reduction in surface area of collagen constructs seeded with 5 × 10^6^ MDCs/ml either in co-culture with motor neurons (solid line) or in monoculture (dashed line). n = 5. Error bars = SEM. * p = 0.03. **d**, **e** 30-µm-thick cross sections were taken from 3D collagen-based constructs seeded with 5 × 10^6^ MDCs/ml and stained for desmin (green) and MAP-2 (red). Note the difference in shape between the rounded construct co-cultured with 1 × 10^6^ motor neurons (**d**) and the elongated MDC-only control (**e**). N.B. The image in **e** represents half the total width of the construct since the entire gel was too large to section reliably. Similar results were obtained across all cultures examined (n = 3). **d**, **e** The widths of the constructs are indicated by red scale bars. These dimensions correspond to those indicated in the macroscale images presented in **a** and **b**. Note that the width in muscle-only constructs is far greater than the orthogonal height, whereas the neuron-muscle co-cultures possess a more rounded morphology. Scale bars = 100 μm.

Due to the high degree of matrix compaction in these constructs at end-point, single contractions of the cultured myotubes could not be detected. In order to characterise any macroscopic differences in levels of matrix compaction between co-cultures and MDC-only controls, images were taken every 7 days during a 3-week culture period and analysed using ImageJ software. The surface area of the construct was used as a measure of matrix remodelling, where a decrease in area correlated with an increase in matrix compaction. Although no difference was observed between co-cultures and MDC-only controls following 7 DIV, a divergence between the two cultures began to emerge by 14 DIV, where the average construct area in co-culture became smaller than that in MDC-only cultures. At 14 DIV, the construct surface area in 3D co-cultures was 222.35 mm^2^ (±25.33) versus 307.54 mm^2^ (±42.24) in MDC-only controls. However, this difference was not significant (p = 0.159). By 21 DIV, however, the difference in average construct surface area was significant (p = 0.03), with co-culture constructs measuring 162.00 mm^2^ (±10.74; n = 5), while MDC-only controls measured 273.08 mm^2^ (±34.44; n = 5) (fig. 4c).

Comparison of co-culture constructs with MDC-only controls showed increased thickening of the collagen matrix along the outside edges of the collagen gel (fig. 4a, b). It is possible that this thickening was a result of increased collagen fibril density due to greater matrix contraction by MDCs. However, it may also have been due to cells within the mixed ventral horn population remodelling the collagen fibrils close to the construct surface and drawing them together. In order to investigate levels of matrix surface compaction by ventral horn cells in co-culture, frozen cross sections were cut from both co-culture and MDC-only controls and immunostained for MAP-2 and desmin (fig. 4d, e). Analysis showed that MDC-only cultures displayed elongated cross-sectional structures with widths far greater than their heights, while MDC-motor neuron co-cultures, by comparison, possessed a cross section far closer to circular. While the captured images provide some evidence for surface compaction of the collagen gels by mixed ventral horn cells, the rounded morphology of the co-culture cross sections, compared with the elongated shape of the MDC-only controls, also suggests an increased level of contraction from within the construct. This result was consistent across all cultures examined (n = 3) and implies that the increased matrix compaction observed in 3D co-culture was due to the action of non-neuronal cells within the mixed ventral horn population and the increase in activity of cultured MDCs. However, the precise mechanism behind this increased compaction could not be determined from the available data.

## Discussion

### Relevance, Applications, and Future Perspectives for Neuromuscular Engineering

A 3D in vitro model of skeletal muscle capable of promoting the formation of robust and functionally mature NMJs between co-cultured myotubes and motor neurons would be of considerable benefit to the continued study of neuromuscular physiology and pathology. Work with organotypic slices and neurospheres has demonstrated the possibility for promoting synaptic development between motor neurons and muscle in 3D culture. However, the use of these neuronal sources carries a degree of ambiguity with regard to which cells are interacting with which. The ability of dissociated neurons to survive and interact with 3D skeletal muscle models is beneficial, since it represents the development of a more fully defined in vitro platform of neuromuscular tissues.

### Discussion of the Presented Data

In the presented original work, motor neurons were found to make up approximately 35% of the cells within the isolated primary ventral horn population, which is similar to numbers reported previously for such cultures [Bloch-Gallego et al., 1991; Kalmar and Greensmith, 2009]. Culture of MDCs in a medium modified from one normally used for the culture of mixed ventral horn cells promoted MDC differentiation and myotube formation, indicating the suitability of this solution for maintaining MDC-motor neuron co-cultures.

Our previous work regarding the development of a 3D collagen-based muscle model has provided evidence for high levels of myoblast alignment and differentiation in these constructs [Smith et al., 2012], producing ordered, parallel arrays of myotubes. MDCs were used instead of purified muscle stem cells in these cultures as fibroblasts are essential for effective collagen remodelling. In vivo, fibroblasts are responsible for producing, organizing, and remodelling the extracellular matrix and, particularly, collagen fibrils [Gillies and Lieber, 2011]. In order to ensure effective matrix restructuring, a mixed population of myoblasts and fibroblasts was, therefore, considered beneficial. This hypothesis is supported by previous work in our group that has shown that purified myoblasts exert weaker remodelling forces and strain development in 3D constructs than mixed myogenic and non-myogenic populations [Brady et al., 2008]. The development of an aligned and anisotropic cellular architecture mimics that seen in the native skeletal muscle tissue (fig. 1b, c), and highlights the suitability of this model for further development towards the production of a more biomimetic skeletal muscle culture system. Furthermore, the ability to maintain densely seeded MDC constructs over many weeks in vitro makes this system well suited to the production of stable co-cultures for long-term analysis.

Immunostaining of muscle-motor neuron 3D constructs demonstrated the ability for these co-cultures to promote regular apposition of pre- and post-synaptic terminals, suggesting the formation of preliminary synaptic contacts between both cell types. As in previous in vitro studies [Bach et al., 2003; Miles et al., 2004; Larkin et al., 2006b; Dhawan et al., 2007; Guo et al., 2011; Smith et al., 2013], co-localisation of pre- and post-synaptic markers in these co-cultures lacked the clear definition of NMJs in vivo [Akaaboune et al., 1999; Sanes and Lichtman, 2001]. This is likely due to the immature nature of the cells utilised for this study, and steps to promote a greater degree of cellular maturation, such as neuronal excitation, may be able to promote the generation of developmentally advanced and robust synaptic contacts between these cell types. Previous work has shown that culture systems such as those described here can support electrical and mechanical stimulation [van der Schaft et al., 2013; Player et al., 2014], meaning that the development and incorporation of muscle maturation stimuli into this model are a credible possibility for future studies. AChRε gene expression has been shown previously to be present specifically in the subsynaptic nuclei of innervated muscle fibres [Brenner et al., 1990; Gundersen et al., 1993]. The up-regulation of mRNA transcripts for this gene in these 3D co-cultures, therefore, suggests a level of maturation within the AChR subunit composition of the cultured myotubes when maintained in close association with supporting motor neurons, providing further evidence for synaptic development and the specialisation of the post-synaptic membrane.

Gene expression analysis from these collagen-based co-cultures highlighted a significant increase in MYH3, MYH8, and troponin T1 mRNA transcripts when compared to MDC-only controls. These data suggest a level of maturation in myotubes co-cultured with motor neurons in 3D through the up-regulation of transcripts for contractile apparatus proteins. However, the modest increase in expression of the adult MYH isoform (MYH1) points toward a relatively immature overall transcription profile, with no evidence of the isoform switching that would accompany muscle maturation during normal embryonic and postnatal development in vivo. The somewhat immature nature of the cultured myotubes is further evidenced by the lack of striations observed in the immunostained cells compared with in vivo counterparts (fig. 1b, c). Despite this immaturity, the high level of construct stability observed in this system provides the possibility to extend culture periods over far longer time courses than is possible in conventional 2D skeletal muscle culture. In this manner, analysis of whether more developmentally mature transcription profiles can be achieved in long-term culture is a viable option for future studies. Previous studies assessing skeletal muscle gene expression provided evidence for a greater degree of adult MYH gene expression in constructs maintained in co-culture with neural tissue [Larkin et al., 2006b]. As stated previously, this study utilised organotypic spinal cord explants as their motor neuron source. The increased muscle maturation reported in the study published by Larkin et al. [2006] may, therefore, be attributable to the presence of supporting cell types and associated tissue structures in the slice preparation rather than the motor neuron presence specifically. Since these structures and/or cell types were absent or present in different ratios in our preparations, the resulting maturation signal may have been weakened. The comparative data indicate that more rigid conformity to tissue level architectures and cell ratios may be required when using dissociated single neurons in order to obtain optimal maturation signals for promoting muscle development.

The use of collagen matrices in this study yielded robust and stable constructs capable of persisting in culture for over 3 weeks. However, extensive compaction of the matrix by fibroblasts led to the formation of rigid structures, which made effective assessment of contractile function difficult. This is not surprising since this model was originally optimised to produce long-term stable muscle constructs for investigating alterations in strain and loading in vitro [Cheema et al., 2005]. Given the primary purpose of this study was to investigate neuromuscular contact in 3D systems and the maturation effect of the motor neuron presence on cultured skeletal muscle myotubes, an inability to measure contraction was not considered a cause for concern. The contractile ability of cells developed within the described culture conditions was verified by visual inspection of cells in conventional 2D culture. Myotubes maintained in medium 2 exhibited spontaneous contractile activity as evidenced by twitching cells (data not shown). However, as already discussed, the lack of striations in the 3D cultured myotubes likely indicates that the generated force from such contractions was relatively weak. The capacity for these collagen constructs to be subjected to mechanical strain regimens [Auluck et al., 2005; Cheema et al., 2005; Player et al., 2014] represents an exciting possibility for the future use of this system and a means to promote myotube maturation to the point where contractile behaviour can be more readily investigated. Furthermore, analysis of NMJ development under different loading and unloading conditions will yield valuable data on the importance of mechanical cues on neuromotor synaptogenesis. For analysis of contractile function, alternative, more flexible matrices should be considered. Studies have shown that supplementation of basal matrices with Matrigel improves the measured contractile function in engineered skeletal muscle constructs, suggesting that manipulating the elastic modulus of engineered matrices by mixing biopolymers enables greater flexibility and, therefore, a greater ability for cultured cells to deform the scaffold [Hinds et al., 2011]. As proof of this, our group's recent study utilising similar nerve-muscle co-culture conditions within a fibrin-based matrix demonstrated the ability for such cells to generate measurable force. Moreover, this study highlighted that co-culture with motor neurons led to a degree of neuronal control over myotube activation, functional maturation, and cytoskeletal organisation [Martin et al., 2015]. While valuable for functional studies, these fibrin constructs were less stable over long-term culture periods and so were ill suited to more extended studies of NMJ maturation in vitro. Our fibrin study and the collagen-based work presented here highlight the importance of selecting appropriate matrix modalities for tissue engineering experiments based on the specific goals of the study.

Significant differences were observed in the level of matrix remodelling in the MDC-motor neuron co-cultures compared with MDC-only controls. After 3 weeks in vitro, co-culture construct surface areas were significantly smaller than those of MDC-only controls, suggesting the level of matrix contraction in co-culture was significantly greater. As described previously, the remodelling observed in these constructs as they develop is thought to correlate with the level of cellular matrix remodelling occurring in culture; the greater the activity of the fibroblasts in culture, the larger the level of bowing observed in the construct. However, myotubes in culture also spontaneously contract, and culture force monitor recordings from mixed myoblast and fibroblast 3D constructs have demonstrated a significantly greater level of overall strain compared with purified fibroblast or myoblast cultures [Brady et al., 2008]. It seems likely that myotube activity would also contribute to the levels of matrix contraction occurring in these 3D constructs, which in turn may be mediated by the addition of a neuronal input, potentially explaining the increased matrix remodelling. Although contraction by non-neuronal cells within the mixed ventral horn population also likely contributed to the observed result, the rounded morphology of the co-culture cross sections, compared with the elongated MDC-only control morphology, suggests greater levels of contraction from MDCs within the collagen matrix.

### Applications of Tissue-Engineered Innervated Skeletal Muscle Models

This work constitutes the first time that dissociated motor neurons have been evaluated in collagen-based 3D co-culture with skeletal muscle cells. The collected data, therefore, provide important proof of principle for the use of such cells in 3D muscle culture and in the development of more defined, complex models of skeletal muscle tissue. Isolated motor neurons are able to survive in this collagen-based 3D skeletal muscle model and are capable of promoting the formation of preliminary neuromuscular contacts whilst maintaining the ordered cellular architecture and maturity of the cultured myotubes. The up-regulation of transcripts for contractile machinery proteins and markers of post-synaptic terminal specialisation suggest that motor neurons promote the maturation of the underlying muscle in these cultures, as is known to occur in vivo.

The establishment of a 3D platform for dissociated motor neuron-muscle co-cultures has obvious potential benefit for the development of disease models incorporating iPSCs. The increasing prevalence of human iPSC technologies since their inception [Takahashi et al., 2007; Yu et al., 2007] now means that the establishment of diseased cell types carrying patient-specific mutations for more accurate recapitulation of a given condition's pathophysiology is a plausible eventuality [Thomson et al., 2012]. This holds exciting promise, particularly for the study of conditions for which no good animal model exists. For example, the standard therapeutic model for ALS is the SOD1G93A mouse model which carries 23 copies of the SOD1G93A transgene [van der Worp et al., 2010]. SOD1 mutations only account for a very small fraction of total ALS cases (∼3%), so such studies are only directly relevant to this subset of patients [Scott et al., 2008; van der Worp et al., 2010]. Furthermore, it has been highlighted that the phenotype of these mice is so exaggerated by the 23 copies of the transgene present that the only pharmacological intervention capable of attenuating the effect is direct inhibition of SOD1 [Scott et al., 2008; van der Worp et al., 2010]. Evidence has been put forward to suggest that these SOD1 mutant mice have a greater susceptibility to infections and other non-ALS-related conditions, and it has been implied that it is this supplementary illness, rather than ALS, that is alleviated by certain experimental treatments. Consistent with this hypothesis, some broad-spectrum antibiotics and general anti-inflammatory agents have been shown to be efficacious in alleviating the pathophysiological phenotype in SOD1G93A mouse models [Scott et al., 2008]. In cases such as this, the possibility of generating cells carrying patient-specific mutations may enable more accurate recapitulation of the disease phenotype, leading to more informative mechanistic studies and predictive novel therapeutic screening applications. Effective models of complex conditions such as ALS have yet to be achieved, but diseased NMJ models have been established for myotonic dystrophy [Marteyn et al., 2011] and spinal muscular atrophy [Yoshida et al., 2015], demonstrating that such model systems are possible.

Compound efficacy/toxicity screening is a second avenue of research for which neuromuscular models could be utilised effectively. Current development protocols can take over a decade and cost over USD 1 billion [DiMasi and Grabowski, 2007]. A substantial reason for this cost is the high rate of attrition of compounds at late development stages, which is in turn due to the inadequacy of preclinical models in terms of predicting drug effects in humans [Esch et al., 2014]. As already discussed, animal models often represent poor surrogates for human systems, and conventional cell culture techniques do little to recreate the structure, maturation, and functional profiles of the native tissue. The generation of biomimetic muscle models with functional innervation could, therefore, prove invaluable to the screening of novel paralytics, muscle relaxants, and treatments for peripheral neuropathic conditions [Beebe et al., 2005]. The generation of multiwell high-throughput contractility assays for skeletal muscle [Vandenburgh et al., 2008, 2010] serves to highlight further the possibility of generating informative and highly predictive screening systems with which to advance therapeutic developments to better suit the needs of patients.

Innervated 3D skeletal muscle models also have the potential for utility in experimental physiology, particularly as a means of investigating the basic biology underpinning NMJ formation. Indeed, in vitro culture models, such as those described here, are amenable to mechanical strain [Player et al., 2014] and electrical stimulation [Khodabukus and Baar, 2012], which are key signals likely governing the development of the neuromuscular system in utero, and as such could be used to understand how these signals alter neuromuscular maturation. Moreover, both mechanical and electrical signals regulate muscle adaptation and phenotype in adults (i.e. exercise/disuse) and as such 3D skeletal muscle constructs with a neural input, which accurately replicate the in vivo niche, could be used to manipulate and further understand the basis of muscle plasticity. Additionally, the ability to combine mechanical and electrical stimuli with exogenous growth factors or putative nutraceuticals in an integrated neuromuscular model would allow for the highly controlled investigation of regulators of skeletal muscle plasticity.

### Future Perspectives

Before tissue-engineered models of innervated skeletal muscle can become viable for widespread adoption, there is a clear need to address issues with maturation. Advanced states of NMJ maturation have been shown in conventional 2D hybrid cultures [Chipman et al., 2014]; however, no such structures have yet been presented in 3D constructs. Methods for maturing constructs in situ must be investigated in order to advance the development of current models toward more biomimetic tissues. Furthermore, little investigation of developing the excitation-contraction coupling machinery has been undertaken in co-cultured cells to date. Although numerous studies highlight the development of pre- and post-synaptic terminals in vitro, the formation of robust t-tubules expressing ryanodine receptors linked to the sarcoplasmic reticulum is essential for complete development of innervated skeletal muscle models. The 3D models presented and discussed here represent ideal platforms in which to investigate how NMJ formation advances the development of the physiologically correct excitation-contraction coupling machinery and, in doing so, drives the maturation of cultured skeletal muscle towards a more biologically relevant phenotype.

It has been shown that repetitive electrical stimulation of motor neuron-myotube cultures for 30–60 min significantly up-regulates synapsin expression and AChR clustering, suggesting such stimuli may be employed to improve NMJ formation in culture [Fukazawa et al., 2013]. This result is logical, given the activity dependence of NMJ structure and development in vivo [Ataman et al., 2008]. Other studies have demonstrated that mechanical stimuli can be used to advance the maturation of skeletal muscle constructs [Player et al., 2014], opening up the possibility for investigating concerted electromechanical conditioning as a means to push innervated skeletal muscle constructs toward a more adult phenotype in vitro.

Other considerations for advancing neuromuscular interactions in vitro are the inclusion of terminal Schwann cells and ‘kranocytes’ (NMJ-capping cells) to better mimic the native structure of synapses [Koirala et al., 2003; Court et al., 2008; Darabid et al., 2014]. Although establishment of triple cell type cultures is daunting, it is possible that inclusion of these cells will provide valuable maturation signals for developing NMJs. Linked to the inclusion of supporting glia in NMJ development is the discovery that, in drosophila, the glia-released Maverick protein activates the transforming growth factor-β (TGF-β) receptor on developing muscle membranes, which in turn leads to the activation of neuronal receptors via a retrograde signalling pathway to help modulate synaptogenesis [Fuentes-Medel et al., 2012]. Similarly, in *Xenopus* studies, TGF-β has been shown to promote agrin up-regulation and subsequent increased AChR clustering [Feng and Ko, 2008]. Given these findings, it may be possible to attain the positive effects of Schwann cell presence in culture without having to incorporate a third cell type, simply through modification of current culture media to include the necessary glial-derived factors (such as TGF-β).

In addition to other NMJ-related cell types, another direction for future research is to investigate the potential integration of innervated muscle models with representations of bone and tendon in order to create more comprehensive analogues of musculoskeletal tissues. As mentioned previously, in vitro models of the myotendinous junction have been published [Larkin et al., 2006a; Kostrominova et al., 2009], and the techniques underpinning the production of tissue-engineered cartilage and bone are similarly well advanced [Chen et al., 2015; Huang et al., 2015; Wu et al., 2015]. Integration of these systems, leading to the generation of a muscle-tendon-bone model, would be extremely beneficial for future studies into musculoskeletal development, response to physiological strain, and breakdown in ageing and disease.

A final consideration is the generation of human iPSC myoblasts for these systems. Almost all models discussed in this paper make use of primary rodent tissue or immortalised cell lines to provide the muscle source. This is likely due to the ease with which such cells can be maintained in culture and the comparative difficulty in maintaining motor neurons. However, in order to recapitulate the human NMJ effectively, especially in case of modelling genetic disorders, techniques for generating human muscle from stem cells must catch up with efforts to generate functional motor neurons. Although some work with human co-cultures has been performed [Guo et al., 2011], the cells used were not iPSCs. The differentiation of myoblasts from iPSCs is not as well established as motor neuron differentiation, but significant progress toward the successful production of human myotubes from these cells has been made [Watanabe et al., 2011; Trokovic et al., 2013; Demestre et al., 2015]. Additionally, improvement in motor neuron differentiation to enable directed production of specific subtypes is also underway [Peljto et al., 2010; Patani et al., 2011]. These advances will be of critical importance to the continued development of more biologically accurate neuromuscular tissue-engineering strategies and likely be instrumental advancing the utility of these models in the near future.

## Conclusions

NMJ engineering holds exciting promise for the development of predictive platforms for disease modelling, drug screening, and other applications. As technologies for manipulating human ESC and iPSC types become more prevalent, the possibilities for generating accurate human models become tantalising. As the data presented here and by others attest, the formation of putative synaptic contacts is possible using a variety of cell types and a number of different platforms. Efforts must now be focused on promoting the maturation of motor neuron-myotube co-cultures in order to generate adult-like tissue structures for effective modelling studies.

## Acknowledgements

This work was funded by a grant provided by the National Centre for the Replacement, Refinement, and Reduction of Animals in Research. A.S.T.S. was supported by an MRC DTA fellowship. L.G. is The Graham Watts Senior Research Fellow, funded by The Brain Research Trust. This activity was conducted under the auspices of the NCSEM England, a collaboration between several universities, NHS trusts, and sporting and public bodies. The views expressed are those of the authors and not necessarily those of NCSEM England or the partners involved.

## Supplementary Material

Supplementary data

Supplementary data
