# Supplementary material for: Creating Interactions between Tissue-Engineered Skeletal Muscle and the Peripheral Nervous System
Source: Cells Tissues Organs. 2016 Nov 9;202(3-4):143–58. doi: 10.1159/000443634 (PMC5175300; doi:10.1159/000443634)
Supplement: Supplementary file 2 — Supplementary data [file cto-0202-0143-s02.docx]

**Creating Interactions Between Tissue-Engineered Skeletal Muscle and the Peripheral Nervous System**

A.S.T Smith^1,3,6^, S. Passey^3,5^, N.R.W. Martin^3^, D.J. Player^3^, V. Mudera^4^, L. Greensmith^1,2†^ & M.P. Lewis^3†^*

*1. Sobell Department of Motor Neuroscience and Movement Disorders, UCL Institute of Neurology UK*

*2. MRC Centre for Neuromuscular Diseases, UCL Institute of Neurology, UK*

*3. Arthritis Research UK Centre for Sport, Exercise and Osteoarthritis, National Centre for Sport and Exercise Medicine (NCSEM) England, School of Sport, Exercise and Health Sciences, Loughborough University, UK*

*4. UCL* *Institute of Orthopaedics and Musculoskeletal Science, Division of Surgery and Interventional Science, UK*

*5. Department of Pharmacology and Therapeutics, University of Melbourne, Australia*

*6. Department of Bioengineering, University of Washington, USA*

^†^Both of these authors are to be credited as Senior Authors of this work

**Supplementary Figure 1.** Effect of motor neuron presence and treatment with Agrin and Wnt3 recombinant proteins on the number of myotubes in 2D culture possessing distinct AChR clusters. n = 4, error bars = standard error of the mean, *p < 0.002.

**Supplementary Table 1:** The composition of Medium 2 used in this study. Volumes given are sufficient to produce 200 mL of medium.

| **Component** | **Volume/ Concentration** | **Source** |
| --- | --- | --- |
| Neurobasal medium | 191 mL | Gibco/ Invitrogen |
| B27 supplement | 4 mL | Gibco/ Invitrogen |
| Horse serum | 4 mL | PAA |
| 2-mercaptoethanol | 100 μL | Gibco/ Invitrogen |
| L-glutamine | 75 μg/ mL | Gibco/ Invitrogen |
| Ciliary neurotrophic factor | 500 pg/ mL | Alomone labs (Jerusalem, Israel) |
| Glial cell line-derived neurotrophic factor | 100 pg/ mL | Alomone labs |
| Brain derived neurotrophic factor | 100 pg/ mL | Alomone labs |
| Insulin-like growth factor-1 | 10 ng/ mL | AbD Serotec, (Oxford, UK) |
| Agrin recombinant protein | 200 ng/ mL | R&D (Abingdon, UK) |
| Wnt3 recombinant protein | 20 μg/ mL | Abnova (Heidelberg, Germany) |

**Supplementary Table 2:** PCR primer sequences used in this study to examine expression of genes associated with muscle maturation and neuromuscular interaction.

| **Gene** | **Forward Primer** | **Reverse Primer** |
| --- | --- | --- |
| RPII | ACATAACGAAGACGGTCAT | TAAGCCATTCAACAAGCAATA |
| Troponin T (slow, type 1) | GTCGGGAGATGAAACTCAGGAT | CAGGTCAAATTTCTCCGATTCC |
| MYH1 (adult fast) | CCTAAAGGCAGACTCTCCCACTGGG | GGCCATCTCGGCGTCGGAAC |
| MYH3 (embryonic) | GCCGGTGTGACTCAGCCAACACTAT | TCCTGGCGCTTTTTGCCTCGG |
| MYH8 (neonatal) | TACGCCAGTGCTGAAGCAGGTA | CCATGGCACCGGGAGTTTTCG |
| AChRε | GGCAGTTTGGAGTGGCCTACGACT | GCAGGACGTTGATAGAGACCGTGC |
